# A preliminary survey of major diseases of ruminants and management practices in Western Tigray province, northern Ethiopia

**DOI:** 10.1186/s12917-018-1621-y

**Published:** 2018-09-26

**Authors:** Getachew Mebrahtu Welay, Dawit Gebremichael Tedla, Gebreyohans Gebru Teklu, Shishay Kahsay Weldearegay, Mearg Belay Shibeshi, Haftom Hadush Kidane, Berhe Beyene Gebrezgiabher, Teklehaymanot Huluf Abraha

**Affiliations:** 1 Department Animal Sciences, College of Agriculture, Aksum University, P.O .Box: 314, Shire Campus, Shire, Ethiopia; 2 School of Geology, College of Agriculture, Aksum University, Shire Campus, Shire, Ethiopia; 3 Department of Biomedical Science, Aksum University, Aksum, Ethiopia; 4 Department of Epidemiology & Biostatistics, School of Public Health, Aksum University, Aksum, Ethiopia; 5 Department of Reproductive Health, School of Public Health, Aksum University, Aksum, Ethiopia

**Keywords:** Disease survey, Management practices, Ruminants, Western Tigray, Northern Ethiopia

## Abstract

**Background:**

Despite the highest population, the productivity of Ethiopian livestock is low, and the direct contribution to the national economy is limited. Poor genetic potential, shortage of feed in quantity and quality, poor health care and management practices are the main contributors to low productivity and production. Data on animal disease and management practices are not in place, in this regard; we estimate the burden of animal health ailments and management practices in ruminants via simple cross-sectional study design in randomly selected peasant associations in western Tigray of northern Ethiopia.

**Results:**

A preliminary disease survey in ruminants and their management practices comprising of cattle, sheep and goats was undertaken to evaluate the existence, burden of ruminant diseases and management practices. A total of 121 randomly selected household respondents of Maikhadrah and Bakhar (26.2%), Dansha (19.7%), Adi-Hirdi (18.0%), Adi-Remets and Inda-Selassie (36.1%) sub-districts were inspected throughout the study period. Most (81%) of farmers feed their animals on free grazing in the open environment travelling from highlands and midlands to lowlands in search of adequate feed (different species of grasses) and crop residues during the wet season up to the beginning of the dry season. Majority of farmers (43.8%) had veterinary access from governmental veterinary officers. Thirty-four (33.9%) of the respondents got veterinary access from illegal drug dealers in mini shops or market. Among the major disease constraints identified; Tick infestation (89.3%), lice infestation (68.6%) mange mite infestation (77.7%) lumpy skin disease (LSD) (42.1%), trypanasomiasis (62.8%) bovine pasteurellosis (52.1) mastitis (13.2%), sheep and goat pox (15.7), abortion (19.0%), dystocia (24.8%), retained fetal membrane (25.6%), prolapsed uterus (13.2%) delayed heat period (38.8%) were most endemic ailments directly affecting livestock production and farmers livelihood.

**Conclusion:**

In conclusion management practices in livestock production is poor to a large extent. Burden and endemicity of livestock diseases are substantially higher. The data obtained could be the source of facts for planners in animal health service delivery system in this sub region.

**Electronic supplementary material:**

The online version of this article (10.1186/s12917-018-1621-y) contains supplementary material, which is available to authorized users.

## Background

Among all the livestock that constitute Ethiopian farm animals, ruminants comprising of cattle, sheep and goats are among the main source of draft power (cattle), wealth accumulation purposes and income generation [1]. Sheep and goat play an important economic role in the overall production system of large and small scale farmers, where most shoat production is for wool, leather and meat production [2].

Ethiopia is endowed with the largest livestock population of an estimated 53.4 million cattle 55.2% are female and 44.8% are males) 23.6 million sheep and 18.6 million goats [3] distributed within the different agro-ecological zones of the nation; about 99% of cattle populations are of local Zebu breed. The remaining 1% of exotic breeds is kept mainly for dairy production in urban and peri-urban areas to fulfill local market milk consumption demands.

Literatures indicate that livestock production and productivity are hindered by poor nutritional value both in quantity and quality, poor livestock husbandry practices, animal health ailments and husbandry constraints [4]. The aforementioned constraints result in subsistence-oriented livestock industry in Africa in general and in Ethiopia in particular [5]. Constraints arising from epidemics and pandemics of animal diseases have been contributed to increased livestock morbidity and mortality too. Consequently; this resulted in reduced livestock production and productivity [6–8].

Livestock husbandry practices are extensively managed on free grazing range/extensive system, specifically in the central part of Ethiopia where crop farming dominates the agricultural practices and with farmers keeping an average of 5 to 15 cows/shoats/household (HH) under the free range system, the animals move about freely to feed on forages/grasses, which are abundantly available during the raining season. Hardly are the animals provided supplementary feeds **(**personal observation**)** and despite poorly designed housing shelter is provide by their owners around their home stead areas to protect from predators [9].

In Ethiopia, where livestock agriculture is merely practiced; cattle are raised in an extensive way in small-holder production systems. As a result of extensive livestock production, productivity is hindered by losses due to animal health problems of which; anthrax, blackleg, lumpy skin disease (LSD),trypanosomiasis and other major health problems experienced are parasites, with liver fluke being the major internal parasites,followed by ticks and biting flies. Animal health is recognized to be significant source of production losses such as low weight gain, draught, fertility and lactation performances **(**Personal observation) [1, 10]. In terms of disease prevention and control, farmers are not fully aware/ hardly take up to veterinary vaccination and treatment center to the affected ruminants as they considered health management as too expensive and distance due to topographical problems to veterinary delivery services; soon after owners are forced taking/treat their animals to ethno-veterinary treatments personally rather than affording modern veterinary medication [11]. Farmers have used traditional medicines and practices to treat their animals for different kinds of livestock diseases alleviation [7].

Major animal health constraints and efficient livestock husbandry practices are yet to be identified and developed respectively. Therefore; the aim of this scenario was to recognize livestock ailments and management practices for further investigation and as decision-making tool for livestock agriculture development. Based on literatures and personal observations from need assessments, it is possible to hypothesize that the management practices in livestock agriculture is poorly practiced resulting in high densities of livestock disease burden.

## Methods

### Description of study design and setting

**Fig. 1:**
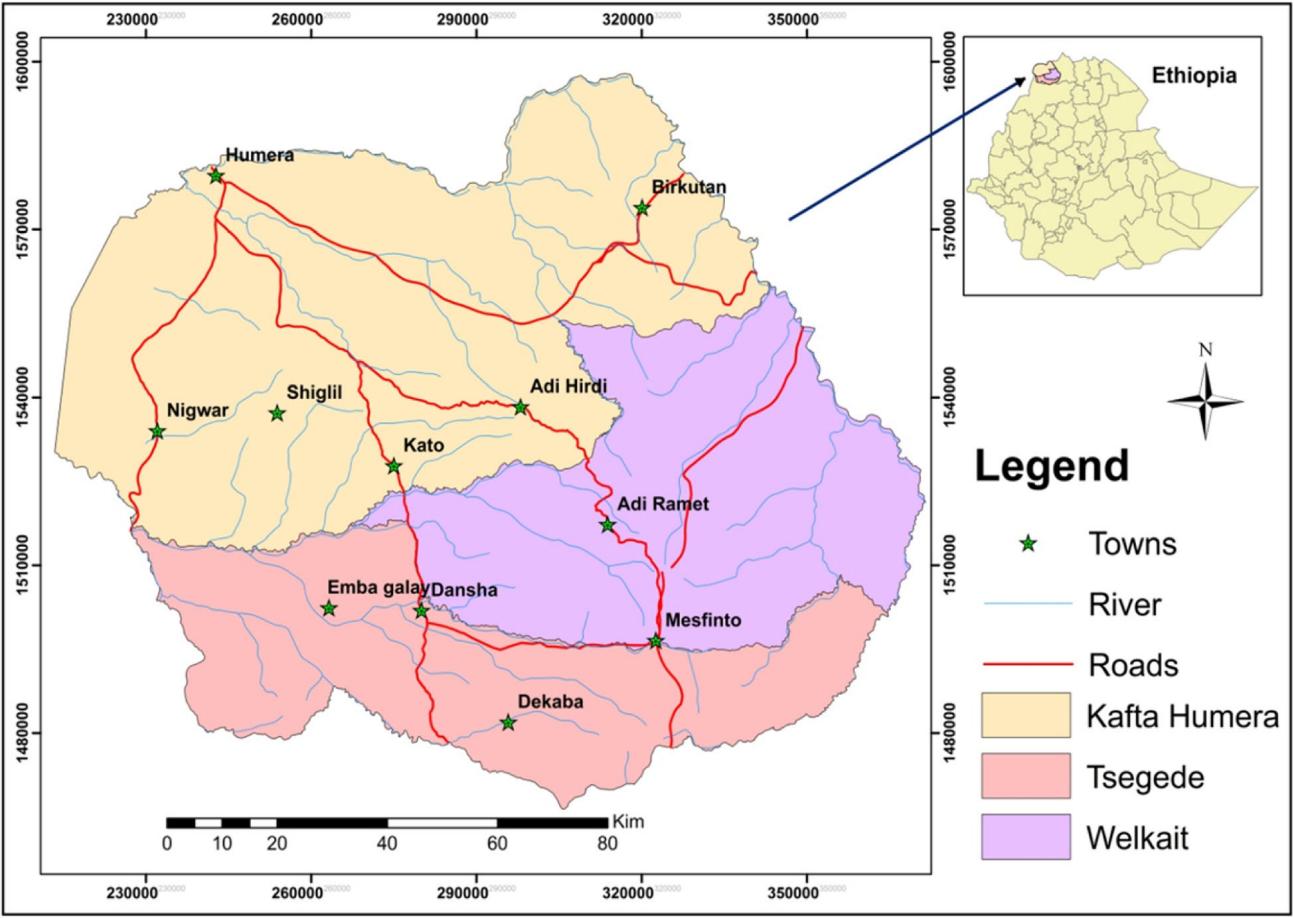
Geographical location of western Tigray province showing the three study districts

A cross-sectional study design was conducted in Western Zone of Tigray, Northern Ethiopia. This area is bordered by Sudan and Eritrea in the west and north respectively and Amhara regional state in the South. The study area includes three districts; Tsegede and Welkayte being highland agro ecological characteristics and lowland characteristics in Kafta-Humera. Six peasant associations were randomly selected per each districts identified as Adi-remets, Adi-hirdi, Inda-selassie, Dansha, Baekhar and Maikhadrah [Fig. 1].

Western Tigray (Humera) positioned at an elevation of 600 m above sea level; is characterized by a very harsh environment with an annual temperature ranging from 27 to 45 °C and rainfall ranges from 900 mm^3^ to 1800 mm^3^ per annum. Humera is one of Ethiopia’s most fertile agricultural zones, with large-scale farming of cash crops such as sesame, maize, cotton, okra, and sorghum [12]. Agriculture is predominantly practiced next to crop agriculture. Local and Begait breeds of cattle, goats and sheep to which a study have been under taken are extensively reared in this sub region (District Agricultural and Rural Development office).

### Data collection method

Simple random sampling technique was applied after dividing districts in to peasant associations and then at household level via multistage sampling procedure. Overall 121 randomly selected respondents comprising of household (HH) farmers, agricultural development agents (ADA) and veterinarians were involved during face to face interview. Structured and semi-structured questionnaire comprising of both qualitative and quantitative data was deliberately developed for this study [Additional file 1] and administered to respondents through *Tigrigna* regional language (farmers) and finally translated in to *English* during data entry and analysis soon after collection of raw data. The questionnaire was pre-tested before administration to the interviewees. Farmers have responded to different animal diseases as they recognize via their grass rooted knowledge of vernacular /local names of different diseases affecting their cattle, sheep and goats on the basis of different disease symptoms and pathognomonic signs. However, animal health personnel’s (Vets and para-vets) from respected veterinary officers were involved during house to house visits to boost further disease classification and identification on the basis of physical examination.

### Data management and statistical analysis

Raw data were entered, edited, checked and managed in to Microsoft Excel Spread Sheet Access. Data organization, summarization and analyses were performed after coding and rechecking, in SPSS Statistics for Windows, Version 20.0.(Armonk, NY:IBM Corp).Simple descriptive analysis was generated (means and proportions, counts and percentage summary statistics. and tests of significance for difference in means and proportions were estimated at 95% confidence interval and 5% (0.05) level of significance was considered as cut point.

## Results

### Housing and feeding system

The mean average number of cattle, sheep and goats per household were 15.58, 11.11 and 13.94 in that order. Mean age at first calving for cattle, sheep and goats was 55.82, 12.03 and 12.37 months respectively. House design for cattle was poorly designed non concrete/natural earthen floor open air around homestead (58%) whereas non concrete fenced with stone wall and wood wall near homestead (45%) housing design was practiced for small ruminants to protect from predators. Most (81%) of farmers feed their animals free grazing from the open environment travelling from highlands and midlands to lowlands in search of adequate feed (different species of grasses) and crop residues during the wet season up to the beginning of the dry season (Table 1).

**Table 1:** Management practices and veterinary delivery services in ruminants, Western Zone of Tigray, Northern Ethiopia (2015–2016)

Management characteristics and practices	Agro ecology		
	Lowland count (%)	Midland count (%)	Highland count (%)
Housing (cattle)			
Open air at grazing areas	24 (42.9)	–	1 (2.6)
Open air around home stead	20 (35.7)	21 (80.8)	29 (74.4)
Fenced	11 (19.6)	3 (11.5)	7 (17.9)
Corrugated iron with stone wall	–	–	–
Thatched roof with mad wall	1 (1.8)	2 (7.7)	2 (5.1)
Housing (Shoats)			
Open air at grazing areas	6 (10.7)	–	–
Open air around home stead	5 (8.9)	2 (7.7)	1 (2.6)
Fenced	19 (33.9)	14 (53.8)	22 (56.4)
Corrugated iron with stone wall	–	–	–
Thatched roof with mad wall	6 (10.7)	2 (7.7)	1 (2.6)
Sesame seed cake	2 (3.6)	–	–
Feeding system/supplementation	2 (3.6)	2 (7.7)	3 (7.7)
Sesame seed cake	2 (3.6)	–	–
Improved forage	2 (3.6)	2 (7.7)	3 (7.7)
Free grazing	38 (67.9)	24 (92.3)	36 (92.3)
Sorghum seed coat	12 (21.4)	–	–
Sesame seed coat	2 (3.6)	–	–
Veterinary service for ruminants when get sick			
Take to clinic	25 (44.6)	12 (46.2)	12 (35.9)
Treat personally with modern medicine	12 (21.4)	1 (3.8)	–
Take to traditional healer	6 (10.7)	4 (15.4)	7 (17.9)
Use personally traditional medicine	12 (21.4)	3 (11.5)	7 (17.9)
Take to clinic & Use personally traditional medicine	1 (1.8)	4 (15.4)	5 (12.8)
Take to clinic & Treat personally with modern medicine	–	2 (7.7)	6 (15.4)
Slaughter	–	–	–
Access to veterinary service			
Governmental veterinary clinic	25 (44.6)	10 (38.5)	18 (46.2)
Private veterinary clinic	7 (12.5)	1 (3.8)	–
Shop or market	17 (30.4)	9 (34.6)	15 (38.5)
Governmental and private	4 (7.1)	5 (19.2)	3 (7.7)
Governmental and shop or market	2 (3.6)	1 (3.8)	3 (7.7)
Governmental, private and shop or market	1 (1.8)	–	–
Distance to nearest veterinary service			
< 1 km	3 (5.6)	1 (3.8)	2 (5.1)
1–5 km	23 (42.6)	8 (30.8)	7 (17.9)
6–10 km	14 (25.9)	7 (26.9)	13 (33.3)
> 10 km	14 (25.9)	10 (38.5)	17 (43.6)
Reasons for loss of ruminants			
Predators	5 (8.9)	–	1 (2.6)
Disease incidents	39 (39.6)	15 (57.7)	20 (51.3)
Poisoning	–	1 (3.8)	4 (10.3)
Drought	4 (7.1)	1 (3.8)	–
Disease and drought	5 (8.9)	7 (26.9)	13 (33.3)
Disease and predator	–	2 (7.7)	1 (2.6)

### Access of veterinary service delivery

Majority of farmers (43.8%) had access for services from governmental veterinary officers. Thirty-four (33.9%) of the respondents got veterinary access from illegal drug dealers in mini shops or market. Nearly 7 % of farmers (6.6%) sought veterinary diagnosis and medication from private veterinary drug venders. In terms of disease prevention and control over their ruminant animals, farmers are not fully aware of taking/hardly take up to veterinary vaccination and treatment centers due to beyond their cost affordability, traditional beliefs that awaiting the sick animal for self-recovery; taking to traditional healer or long distance reasons. The nearest distance to veterinary service delivery center ranges from one kilometer (5%) to beyond ten kilometers (34.5%). Forty two percent of respondents (42.1%) could afford the cost for vaccination and treatment; nearly 10 % of the farmers (10.7%) treat personally with modern medicine; 14 % (14%) of them take their sick animal to traditional healers and 18 % of the respondents (18.2%) treated their sick ruminants personally from traditional and herbal medicines.

### Main factors for livestock losses and diseases of ruminants

Livestock diseases are significant challenges to the livestock production sub-sector in Ethiopia. Main risk factors for livestock losses in the study sub-region were disease (61.2%), predators (5%), drought (4.1%), poisoning/grass toxicosis (4.1%) and theft (2.5%). Morbidity rates of cattle, sheep and goats were determined as 8, 12.5, and 15.5% in that order. Ninety one percent of all ailments identified were communicable diseases consisting of bacterial, viral, protozoal and parasitic infections.

The major ruminant disease constraints limiting livestock production in the study setting were lumpy skin disease (LSD) (42.1%), trypanasomiasis (62.8%) bovine pasteurellosis (52.1) mastitis (13.2%), sheep and goat pox (15.7%) foot rot (11.7%). Among the reproductive disorders; abortion (19.0%), dystocia (24.8%), retained fetal membrane (25.6%), uterine prolapsed (13.2%) delayed heat period/prolonged anestrous (38.8%) were the ailments identified. Grass toxicosis (21.5%), bloat (18.2%) and hyena byte (8.3%) were among the non-communicable ailments and injuries identified. Parasitic diseases of ticks (89.3%), lice (68.6%) and mange mite (77.7%) infestation were among the devastating health problems in livestock production system. Detail burden and frequency of endemic diseases are reported in agro ecological zone wise in (Table 2).

**Table 2:** A preliminary survey of major diseases of ruminants Western Zone of Tigray, Northern Ethiopia (2015–2016)

Disease characteristics	Vernacular names	Agro ecology			Ethno-veterinary medicine Practices
		Lowland count (%)	Midland count (%)	Highland count (%)	
Tick infestation	Kurdid	53 (94.6)	21 (80.8)	34 (87.2)	Topical lotion with kerosene, picking manually
Lice infestation	Kumal, Kunchi-gobay	35 (62.5)	18 (69.2)	30 (76.9)	Washing with ash
Flea infestation	Kunchi	36 (64.3)	8 (30.8)	9 (23.1)	Dipping in ponds Cutting, shearing, clipping the fur
Biting Flies	Nihbay, Karma	35 (62.5)	10 (38.5)	7 (17.9)	–
Biting Birds	Chirna’e	12 (21.4)	___	___	Chasing from the back, Look after the back of their cattle against biting birds.
Mange mites	Abek, Shuhur, Juri, Hafew	43 (76.8)	23 (88.5)	28 (71.8)	Salt + butter (topical lotion)
Fasciolosis	Kaba				
Sheep Kid	Kimanjer	11 (19.6)	___	6 (15.4)	
LSD	Enfurur, Kubkubta, Togtogta, Shlimiye	27 (48.2)	10 (38.5)	15 (38.5)	Coffee ceremonial practices, smoking
GIT parasitosis	Wesfat, Gondera	29 (51.8)	13 (50.0)	13 (33.3)	Drenching with sagla (leaf)
Trypanosomosis	Silim	50 (89.3)	18 (69.2)	8 (20.5)	
Grass toxicosis	Afel, Efel, Maget	5 (8.9)	5 (19.2)	16 (41.0)	Drenching with yoghurt, Drenching with yoghurt, detergent and sesame oil.
Ovine pasteurellosis	Mieta, Bueta	19 (33.9)	3 (11.5)	3 (7.7)	Incision at facial artery
Caprine-pasteurollosis	Mieta, Bueta	23 (41.1)	10 (38.5)	11 (28.2)	Incision at facial artery
Bovine- pasteurellosis	Yelam-geta, Halafiyien, Hangofta, Shuwta, Kofa	27 (48.2)	15 (57.7)	21 (53.8)	Incision, Cauterization, Withholding water for 2–3 days
Anthrax	Megerem, Nefri	14 (25.0)	8 (30.8)	8 (20.5)	Cauterization
Lungworm	Se’al	2 (3.6)	6 (23.1)	5 (12.8)	–
Arthritis	Kirtimat	4 (7.3)	2 (7.7)	1 (2.6)	Incising,cauterization
Ascites	Himam-kebdi, Wel’e	0 (0.0)	5 (19.2)	8 (20.5)	Cauterization
Shoat pox	Infurur, Shilime	12 (21.4)	2 (7.7)	5 (12.5)	Smoking, coffee ceremonial
Paramphistomiasis	Keyahti-hasaku	6 (10.6)	–	5 (12.8)	–
Rabies	Himam-Ebudkelbi	–	6 (23.1)	4 (10.3)	Drinking whey (for humans)
Delayed heat period	Awra-gedib	3 (5.4)	15 (57.7)	29 (74.4)	–
Leech infestation	Alekti	–	–	4 (10.3)	Nasal drenching with tobacco leaf
Foreign bodies	Lastic	1 (1.8)	3 (11.5)	2 (5.1)	–
Blackleg	Weke’e	9 (16.1)	3 (11.5)	3 (7.7)	Drenching with shenfa’e,cauterization
Bovine Tuberculosis	Menkersa, Zigag	11 (19.6)	–	–	Cauterization,dusting.
Parafilariasis		–	–	4 (10.3)	Lotion with honey
Listroiosis	Azurit	–	–	2 (5.1)	
Actinobacilosis	Tokiba	5 (8.9)	–	5 (12.8)	Topical lotion with Butter +Charcoal
Brucellosis	Aber’e	3 (5.4)	5 (19.2)	1 (2.6)	–
Foot rot	Finfin, Hinkasse	8 (16.3)	–	3 (11.5)	Salting
Dermatophilosis	Urit	2 (3.6)	–	2 (5.1)	–
Cow mastitis	Himam-Tub	13 (23.2)	–	2 (5.1)	Cauterization
Contagious Bovine pleuropneumonia (CBPP)	Weke’e–samba, Samba-michi	11 (20.0)	–	5 (12.8)	–
Orf	Kafay	11 (30.6)	4 (25.0)	3 (12.5)	Water (H_2_O) + Salt (NaCl)
Pest petis des ruminants (PPR)	Wetetie, Kur	12 (32.4)	4 (25.0)	3 (13.0)	–
Abortion	Chinga’fe, Aber’e	17 (30.4)	3 (11.5)	3 (7.7)	–
Contagious caprinepleuro pneumonia	Sanabu’e Samba-michi	3 (7.9)	–	2 (8.0)	–
Dystocia	Himam-Hirsi	25 (44.6)	2 (7.7)	3 (7.7)	Manual removal
Cysticercosis	Koso	–	–	1 (2.6)	–
Hydatidosis	–	2 (3.6)	–	1 (2.6)	–
Tunga-penetrans	Mojeliye	3 (5.4)	–	1 (2.6)	Manual removal, salting
Retained fetal membrane	Meskab	22 (39.3)	4 (15.4)	5 (12.8)	Local plant medicine+H_2_O (drenched)
Uterine prolapsed	Mahtsen Miglbat	11 (19.6)	3 (11.5)	2 (5.1)	Manual intervention
Bloat	Menfahti	3 (5.4)	5 (19.2)	14 (35.9)	Drenching local malt, food oil, caticala/uozo and red peppers(powder), Puncturing(rumen)
Malignant tumor	Mandaeti	1 (1.8)	1 (3.8)	0 (0.0)	–
Phimosis	–	1 (1.8)	2 (7.7)	0 (0.0)	–
Paraphimosis	–	1 (1.8)	1 (3.8)	1 (2.6)	–
Actinomycosis	Zigag	6 (10.7)	2 (7.7)	1 (2.6)	Puncturing, Cauterization
Abscess	Hibat	4 (7.1)	2 (7.7)	2 (5.1)	Incising, cauterization
Metritits	Raksi-mahtsen	9 (16.1)	–	–	–
Thelaziasis	Hasaka-Ayeni	2 (3.6)	3 (11.5)	1 (2.6)	Manual drawing out
Bottle Jaw	–	1 (1.8)	–	3 (7.7)	–
Hyena bite	Zibe’e, Arawit	4 (7.1)	4 (15.4)	2 (5.1)	Slaughtering
Warts/skin cancers	Tuba-adgi	2 (3.6)	5 (19.2)	–	Cauterization, burning

## Discussions

This is the first survey narrating in livestock disease distribution and management practices ever undertaken in the field of animal production system. The study also describes the veterinary service delivery system in this sub-region western Tigray of Ethiopia.

Our study findings show a remarkable poor livestock management system and high disease burden. Breeding performance in our local breeds of cattle is very low, characterized by delayed age at first calving. Descriptive analysis show that the mean age at first calving for all ruminants is too long; this dalliance is due to the attribute of poor livestock management and handling practices from local livestock breeders and high incidence of animal diseases. Poor fertility and low genetic potential are contributing factors too [13]. There is a significant difference in mean age at first calving among the lowland, midland and highland across geographical locations. Mean time to first calving in lowlands is a lesser amount as compared to highland and midland ecological locations owing to high availability of feed from agricultural byproducts and grasses.

Livestock breeders are accustomed to feed their livestock free grazing type of feeding system from the locally available feed combinations of grasses, crop residues and crop aftermath. Livestock feeding system is characterized by inadequate provision of concentrate supplementation during the dry seasons when feeds are scarce. This arises from the fact that farmers prefer large number of ruminants in quantity that cannot afford feed and water at zero grazing level. This could be a leading grass rooted cause of low livestock production, productivity and susceptibility to abundant disease ailments [14, 15].

From the study, most frequently, it was observed that the major causes of livestock losses were due to disease, predators, drought, grass toxicosis and theft. This finding is lower in proportion in comparison to previous reports [14, 16]. In majority of farmers, large ruminants are housed in poorly designed pens around homestead or at grazing areas in the open environment where feed and water are accessible as the farmers travelling via their family labor along with their cattle due to limited grazing areas from highlands and midlands to lowlands in search of adequate feed with different species of grasses and crop residues during the wet season up to the beginning of the dry season.

Greater parts of farmers have been getting access to veterinary services from governmental animal health centers. Although most farmers have got access from the aforementioned governmental institution, their intention to use private veterinary services and local traditional medicines is in a very demand. Thirty-four percent of the respondents got veterinary services from black market and illegal drug dealers in mini shops. Nearly 7 % of farmers sought veterinary diagnosis and medication from private veterinary clinic/pharmacy which is lower than a report from Kagira and Kanyariin Kisumu municipality of Kenya [15]. This constraint is due to the fact that the allocation of veterinary service delivery system is low; the quota is one veterinary clinic per three peasant associations. In terms of disease prevention and control over their ruminant animals, farmers are not fully aware of taking/rarely take up to veterinary vaccination and treatment centers due to different challenges; traditional beliefs that awaiting the sick animal for self-recovery; unable to afford medicines, taking to traditional healer, treat their animals personally or due to long distance to veterinary service delivery centers [14, 17].

During face to face interview, quantitative data were triangulated with qualitative data why most farmers are not taking their animals to veterinary service center prefer taking to other options. Their reply was the following statements;

*“I am too busy to take my patient animals because of crop agricultural works during sawing to harvest.”*

“ *Outpatient treatment follow up due to long distance is tedious”*

*“Traditional medicines are more effective than modern medicines”.*

*“Animal health practitioners do not inject the patient animal at site of swelling but in the intramuscular muscle”.*

This is because farmers prefer their ruminants get injected at the site of swelling to intramuscular injection; considering as if it were rapid treatment response*.*

Ethno-veterinary medicine deals with people’s knowledge, skills, methods, practices and beliefs about the care of their animals [11, 18]. Farmers in various regions of the world still use ethno-veterinary medicines and practices to treat and alleviate infectious and non infectious ailments [9, 11]. Ethno veterinary medicines and practices often provide cheaper options than comparable modern drugs. Furthermore, ethno-veterinary medicine lends itself easily to local adaptation and application [18].

In this regard; our finding indicate that majority of livestock breeders use ethno-medicines and traditional practices like what farmers in various parts of the world do despite most breeders in this locality could afford the cost for vaccination and treatment. From this analysis nearly 10 % of the farmers treat personally with modern medicine; 14 % of them take their sick animal to traditional healers and 18 % of the respondents treated their sick ruminants personally from traditional practices and herbal medicines. These figures verify that Livestock owners could not rely on veterinary services only for control and prevention of various important livestock diseases; they need to develop socially acceptable and effective remedies from reasonably inexpensive sources that can complement modern medicines. The indigenous knowledge of Ethno veterinary medicine provides such an opportunity for livestock health care practices which are cheap and locally available than pharmacotherapy. We found Farmers can prepare and use homemade remedies without any expenditure [19, 20].

Among these number of infectious ailments reported in this current study; ectoparasitic infestation were found inflicting severe damages in ruminants [7]. This attribute is because of poor livestock handling practices; animals are freely grazing mixed with other non accaracide treated ruminants in rangelands and pastures [15]. Livestock owners seldom use modern anti-parasitic formulations; diazinon and accaracides and management practices to treat their cattle and shoats to treat against arthropod parasites (personal observation). Bacterial and viral infections are highly reported in our settings. This is more probably due to the fact that our study region is bordered by transboundry livestock disease prone ecological regions of Sudan, Eritrea and Amhara regional state in the West, North and South in that order [19]. In the context of our findings, livestock infections and infestations were being found as global livestock diseases important to the poor in line with the 2016 report of Office International Des Epizootics (OIE) [21].

### Limitations

The study relies on participants’ response rates, as a consequence; there might be under and/or over estimation of reporting in disease prevalence. Therefore, the estimated prevalence may not exactly show the exact burden of each disease in the study settings. Moreover, self report of ruminant diseases by farmers was used during major disease surveillances. Hence, recall bias could have been existed.

## Conclusions

In conclusion the distribution of livestock diseases is substantially higher. Management practices in livestock production are poorly practiced. This is a greatest threat for livestock production and productivity. More fundamentally, investment in animal infections and infestations control and management practice strategies is necessary to reduce the multiple impacts of livestock diseases and deaths on animal health and production. The poor fertility and poor livestock performance can be addressed through better management and crossbreeding with improved breeds.

## Additional file

Additional file 1: Questionnaire. (DOCX 23 kb)

## Abbreviations

ADA: Agricultural development agents
CBPP: Contagious bovine pleuropneumonia
GIT: Gastro intestinal tract
HH: Household
LSD: Lumpy skin disease
OIE: Office International Des Epizootics
PPR: Pest petis des ruminants
