# Supplementary material for: A preliminary survey of major diseases of ruminants and management practices in Western Tigray province, northern Ethiopia
Source: BMC Vet Res. 2018 Sep 26;14:293. doi: 10.1186/s12917-018-1621-y (PMC6158858; doi:10.1186/s12917-018-1621-y)
Supplement: Supplementary file 1 — Questionnaire. (DOCX 23 kb) [file 12917_2018_1621_MOESM1_ESM.docx]

**Tigray Agricultural Research Institute (TARI)**

**Humera Agricultural Research Center (HuARC), Livestock Research Team**

**Data collection format (Questionnaire)**

**I. General information**

1. Administrative zone_____________district__________peasant association _____ household number_________

2. Agro ecological zone (circle one) 1. Low land 2. Mid land 3. High land

3. Name of respondent_______________

4. Age of respondent_______________

5. Sex of respondent________________

**II. Livestock Production system**

1. Type of production system
   1. Extensive production system
   2. Semi- extensive production system
   3. Intensive production system
   4. Specify others (if any) ­­­­­­­_______________
2. Type of commodities: 2.1 Cattle 2.2 Sheep 2.3 Goats
3. Number of total commodities per household cattle________ Sheep______ Goats______

**III Reproduction performance of Ruminants**

1. Age at first mating for cattle _______ sheep________ goat________
2. Age at first calving/lambing/ kidding cattle_______sheep_______ goat ______
3. Number of lifetime parities cattle_______ sheep _______ goat__________
4. Mating system 4.1 Natural 4.2 Artificial Insemination(AI)

**IV. Feeding system**

1. Do you practice feed selection? 1. Yes 2. No
2. Do you practice feed supplementation? 1. Yes 2. No
3. If yes what are the types of feed supplementation?

1. Sesame seed cake

2. Improved forage

3. Free grazing

4. Sorghum seed coat

5. Sesame seed coat

**V. Housing system**

1. House design for cattle (circle)

1. Open air at grazing areas

2. Open air around home stead

3. Fenced

4. Corrugated iron with stone wall

5. Thatched roof with mad wall

2. Housing design for sheep and goat (shoats)

1. Open air at grazing areas

2. Open air around home stead

3. Fenced

4. Corrugated iron with stone wall

5. Thatched roof with mad wall

**VI. Veterinary services**

1. Veterinary service for ruminants when get sick
2. Take to clinic
3. Treat personally with modern medicine
4. Take to traditional healer
5. Use personally traditional medicine
6. Take to clinic & Use personally traditional medicine
7. Take to clinic & Treat personally with modern medicine
8. Slaughter
9. Do nothing with
10. Access to veterinary service

1. Governmental veterinary clinic
2. Private veterinary clinic
3. Shop or market
4. Governmental and private
5. Governmental and shop or market
6. Governmental, private and shop or market

3. Distance to nearest veterinary service

1. <1km 2. 1-5 km 3. 6-10 km 4. > 10 km

4. Reasons for loss of ruminants

1. Predators

2. Disease incidents

3. Poisoning

4. Drought

5. Disease and drought

6. Disease and predator

5. Why don’t you take your ruminants to the veterinary clinic?

________________________________________________________________________________________________________________________________________________________________________________________________________________________________________________________________________________________________________________________

6. Why do you practice traditional medicines in place of modern me

____________________________________________________________________________________________________________________________________________

______________________________________________________________________

**VII. Animal Health**

1. Rank common external parasites in ruminants and their traditional treatments

| Serial .No_ | Cattle | sheep | goat | Vernacular name | Traditional treatment |
| --- | --- | --- | --- | --- | --- |
|  |  |  |  |  |  |
|  |  |  |  |  |  |
|  |  |  |  |  |  |
|  |  |  |  |  |  |
|  |  |  |  |  |  |
|  |  |  |  |  |  |
|  |  |  |  |  |  |

1. Rank common internal parasites in ruminants and their traditional treatments

| Serial .No_ | cattle | sheep | goat | Local name of disease | Traditional treatment |
| --- | --- | --- | --- | --- | --- |
|  |  |  |  |  |  |
|  |  |  |  |  |  |
|  |  |  |  |  |  |
|  |  |  |  |  |  |
|  |  |  |  |  |  |

1. List and rank endemic livestock diseases( animal health constraints) in order of morbidity and mortality

| Serial .No_ | cattle | sheep | goat | Local name of disease | Traditional name of disease |
| --- | --- | --- | --- | --- | --- |
|  |  |  |  |  |  |
|  |  |  |  |  |  |
|  |  |  |  |  |  |
|  |  |  |  |  |  |
|  |  |  |  |  |  |
|  |  |  |  |  |  |
|  |  |  |  |  |  |
|  |  |  |  |  |  |
|  |  |  |  |  |  |
|  |  |  |  |  |  |
|  |  |  |  |  |  |
|  |  |  |  |  |  |
|  |  |  |  |  |  |
|  |  |  |  |  |  |
|  |  |  |  |  |  |

1. What are the most common cause of morbidity and mortality

1. Infectious 2. Non infectious

5. Encircle any reproductive disorders based on their frequency and severity

1. Retained fetal membrane 6. Metritis

2. Dystcia 7. Abortion

3. Uterine prolapsed 8. Still birth

4. Vaginal prolapsed 9.Phymosis and paraphymosis (males)

6. List and rank any injury and accident causes of death of your ruminants

1. _____________________

2._______________________

3._______________________

4.______________________

5.______________________

**Thank you so much!!!**
